# Validity and reliability of the Difficulties in Emotion Regulation Scale Short Form in Indonesian non-clinical population

**DOI:** 10.3389/fpsyt.2024.1380354

**Published:** 2024-03-25

**Authors:** Febrianti Santiardi Danasasmita, Veranita Pandia, Efi Fitriana, Irvan Afriandi, Fredrick Dermawan Purba, Abdullah Ichsan, Kent Pradana, Alfonso Haris Setia Santoso, Fithriani Salma Mardhiyah, Rita Engellia

**Affiliations:** ^1^ Department of Psychiatry, Faculty of Medicine, Universitas Padjajaran, Hasan Sadikin Hospital, Bandung, Indonesia; ^2^ Department of Psychology, Faculty of Psychology, Universitas Padjadjaran, Bandung, Indonesia; ^3^ Center for Psychometric Studies, Faculty of Psychology, Universitas Padjadjaran, Bandung, Indonesia; ^4^ Department of Public Health, Faculty of Medicine, Universitas Padjadjaran, Bandung, Indonesia; ^5^ Center for Psychological Innovation and Research, Faculty of Psychology, Universitas Padjadjaran, Bandung, Indonesia

**Keywords:** emotion regulation, adolescents, young adult, validation, reliability, psychometric properties, Indonesia

## Abstract

**Introduction:**

Emotion dysregulation is a transdiagnostic marker for vulnerability and has high comorbidity rates across various range of disorders among adolescents and young adults, highlighting the crucial need for precise assessment tools to recognize its significant impact on well-being. The Difficulties in Emotion Regulation Scale Short Form (DERS-SF) is a comprehensive instrument designed to measure the ability to regulate emotion. This study aimed to investigate the psychometric properties of DERS-SF among the non-clinical population, particularly high school and university students in Indonesia.

**Methods:**

A total of 738 senior high school and university students completed the Indonesian version of DERS-SF and standard questionnaires to assess its validity, consisting of the Depression Anxiety Stress Scale (DASS), the Beck Depression Inventory-II (BDI-II) for young adults and the Children Depression Inventory (CDI) for adolescents. Three models were examined in factorial validity tests using confirmatory factor analysis.

**Results:**

The results showed that DERS-SF had an overall good internal consistency with Cronbach’s alpha coefficient of.89 for the 18-item version,.90 for the 17-item version, and.91 for the 15-item version. Test-retest reliability was moderate with a value of.67. In addition, it had good satisfactory content as shown by item content validity index (I-CVI) = .96 and scale content validity index (S-CVI) = .83, as well as convergent validity. All subscales scores showed a positive and strong correlation with DASS, BDI-II, and CDI except awareness. Based on confirmatory factor analysis (CFA), the correlated 6-factor model excluding item number 6, and the 5-factor model excluding awareness were suitable to use in non-clinical populations.

**Conclusion:**

This study established the removal of the awareness subscale in the DERS-SF Indonesian version, resulting in better reliability and validity than the original version with complete subscales.

## 1 Introduction

Emotion is recognized for playing a central role in the human experience but defining the concept precisely is challenging and frequently debated. Based on previous reports, emotion is made up of dedicated neural circuits, response systems, as well as a feeling state/process that motivates and organizes cognition and action (1, 2). It also provides information to the person concerned and can include previous cognitive appraisals as well as ongoing cognition, including an interpretation of feeling states, expressions, or social-communicative signals. Moreover, emotion can motivate approach or avoidant behavior, exercise control/regulation of responses, and be social or relational in nature (3). Understanding the complexities of emotion and its regulation is essential for comprehending the nuances of human behavior and mental well-being.

Emotion regulation is the process of managing and modifying emotional responses to achieve a desired outcome. Some theories of emotion regulation focus on controlling experience and expression, particularly the expressive control of negative emotion as well as reducing emotional arousal. Meanwhile, others underscore the functional nature of emotion when conceptualizing regulation, suggesting it is not always synonymous with immediate reduction of negative affect (4). Thompson (1994) defines emotion regulation as “the extrinsic and intrinsic processes responsible for monitoring, evaluating, and modifying emotional responses, particularly the intense and temporal characteristics, to achieve individual goals” (5). Furthermore, emotion regulation is defined by Gross (1998) as a set of mental techniques that people employ to either suppress, amplify, or sustain feelings based on the situation and desired outcome (6). The concept of emotion regulation underscores the importance of accepting and valuing responses rather than eliminating certain emotion (4). This concept is a critical affective mechanism for psychological well-being (7, 8). People at risk of mental illness might benefit from efficient emotion regulation (9).

On the other hand, emotional dysregulation involves experiencing and expressing emotions in an overly intense, unstable, rigid, or prolonged way, hindering effective interpersonal interactions or goal oriented behavior (4, 10, 11). Furthermore, emotional dysregulation is a transdiagnostic indicator for vulnerability and high rates of comorbidities in various diagnoses (7, 8). It is associated with psychopathology in a variety of neuropsychiatric illnesses, including borderline personality, generalized anxiety, substance abuse, and posttraumatic stress disorder (12). The broad role of emotional dysregulation has offered new dimensional representations of psychopathologies in recent studies, with a crucial goal of enhancing emotion regulation capacities (12). Recognizing the significant impact of emotional dysregulation on mental health emphasize the need for accurate and comprehensive measurement tools.

The Difficulties in Emotion Regulation Scale (DERS) is one of the most commonly used measurement for emotion regulation. It was developed more comprehensively than existing questionnaires to measure emotion dysregulation (4). The evaluation of multiple domains of emotion dysregulation, including cognitive, affective, and behavioral, was carried out using an acceptance-based theoretical framework (13). The DERS items were selected to demonstrate issues in the following dimensions of emotion regulation: (a) awareness and understanding; (b) acceptance; (c) the ability to engage in goal-directed behavior and refrain from impulsive behavior when experiencing negative emotion; and (d) access to effective regulation strategies (4).

The first version of DERS consists of 36 questions divided into six dimensions: non-acceptance, goals, impulses, awareness, strategies, and clarity (4). *Non-acceptance of emotional responses* (non-acceptance) shows a proclivity for negative secondary reactions to emotion and/or denial of distress. Another subscale, *the difficulties engaging in goal-directed behavior* (goals), captures the difficulties concentrating and completing tasks while experiencing negative emotion. *The impulse control difficulties* subscale (impulse) reflects difficulties controlling behavior when upset. *The lack of emotional awareness* (awareness) subscale measures inattention to emotional responses. *The limited access to emotion regulation strategies* (strategies) subscale assesses beliefs that an individual is restricted in effectively regulating emotion after becoming upset. Finally, *the lack of emotional clarity* (clarity) subscale reflects the degree to which individuals are unsure about their emotions (4, 7).

There are three briefer versions, namely DERS-16 (14), DERS-SF (7), DERS-18 (15). Two versions (DERS-SF and DERS-18) retained all the subscales and one (DERS-16) did not. The awareness subscales were removed from DERS-16, but this did not result in better concurrent validity from either DERS-SF or DERS-18. Both DERS-SF and DERS-18 awareness subscales were substantially related to depression, supporting the retention. Although the brief scales are new and yet to be widely used, DERS-SF has been in use longer and is cited more frequently than the DERS-18 (15, 16). This scale may be useful in future measurement and comparison with results from the largest number of studies (9).

DERS-SF was developed by Kaufman et al. (7), who proposed an 18-item questionnaire with the retention of its original subscales. It has good psychometric properties, including adequate reliability with Cronbach’s alphas ranging from.79 to.91, and concurrent validity in samples of adults and adolescents aged between 12 and 18 years from the United States. Furthermore, correlations between the DERS and DERS-SF subscales varied from.91 to.98, showing that the short and original versions of the DERS shared 83% to 96% of the variation (7). Different subscales were associated with distinct clinical issues; for example, clarity and strategies subscales were linked to various clinical outcomes. The awareness subscale was significantly associated with depression. Meanwhile, the non-acceptance subscale was linked with borderline personality disorder symptoms (9).

Since its development, DERS-SF has been translated and validated into several non-English languages, such as Spanish (17), Finnish (18), Portuguese (17), and Italian (19). However, studies about DERS-SF validation in Indonesia were limited in the literature.

This study aimed to adapt the Difficulties in Emotion Regulation Scale Short Form (DERS-SF) into Bahasa Indonesia to test the reliability, content validity, convergent validity, and confirm the factorial structure in a large sample of Indonesian students. The validated questionnaire might be used in the early detection of emotion dysregulation to develop strategies to prevent mental disorders, particularly for students and the general population. The clinical and scientific utility of DERS-SF has a promising future with an ever-increasing focus on mental health and well-being, specifically in developing countries such as Indonesia.

## 2 Materials and methods

### 2.1 Participants

This study used a cross-sectional design comprising 342 (46.3%) senior high and 396 (53.7%) university students in Bandung, West Java Province, Indonesia. Data were collected using consecutive random sampling from January to April 2021. The age range of the participants was from 15 to 29 with a mean and standard deviation of 18.79 and 1.87. The majority of participants were female (76.9%).

### 2.2 Instruments

To evaluate emotional regulation, symptoms of anxiety, depression, and psychological distress of participants, the following self-report tests were given.

#### 2.2.1 DERS-SF

DERS-SF is an 18-item self-administered questionnaire to measure a person’s ability to regulate emotion and be flexible (4). The original DERS was developed by Gratz and Roemer (2004), consisting of 36 questions based on six subscales. The subscales include non-acceptance, goals, impulse, awareness, strategies, and clarity. DERS-SF is a briefer version of DERS that retains the original six subscales, consisting of 3 items for each. Each answer option ranges from 1 to 5, where 1 = almost never, 2 = sometimes, 3 = about half the time, 4 = most of the time, and 5 = almost always. DERS total score are combined from scoring all items except the awareness subscale, which includes reversal coding in item 1, 4, and 6. Higher total scores indicate more difficulties in emotion regulation (20).

Each subscale from the DERS-SF had Cronbach’s alpha coefficients exceeding.70, ranging from.79 to.91. The DERS-SF values were comparable to the original DERS. Correlations between the DERS and DERS-SF subscales were calculated to determine their similarity for participants. Despite a significant reduction in items, all correlations were above.90 and ranged from.91 to.98, indicating that the DERS-SF and original DERS scales shared 83-96% of their variance (7).

#### 2.2.2 The Beck Depression Inventory-II

The BDI-II is a self-administered 21-item questionnaire that assesses the severity of depression symptoms. This measure was selected as another significant index of concurrent validity for DERS-SF since emotion dysregulation was associated with the risk for depression (21, 22). Participants must rate each item using one of four response options based on the severity of symptoms experienced in the previous weeks, ranging from no to severe symptoms. Each response option was evaluated on a scale of 0 (no) to 3 (yes) (severe). The BDI-II is divided into three subscales namely cognitive, somatic, and affective. Beck et al. and Wang & Gorenstein conducted a study using the BDI-II in different languages, with a mean Cronbach’s alpha of.90 and a range of.83 to.96 (23, 24). The BDI-II Indonesian version had been approved (25), with Cronbach’s alpha coefficient of.94 and only university students completed the scale.

#### 2.2.3 The Children’s Depression Inventory

The CDI is a 27-item questionnaire that examines depression symptoms in children and adolescents during the previous two weeks (26). This scale is standardized for the Indonesian population by Widhiarso et al. (27). This measure was chosen as another important indicator of concurrent validity for DERS-SF because emotion dysregulation has been associated with an increased risk of depression. The five subscales include negative mood, interpersonal difficulties, ineffectiveness, anhedonia, and negative self-esteem. CDI has been shown to be a reliable method for detecting depressive symptoms in both Western (28–30) and Asian populations (27, 31, 32). Cronbach’s alpha coefficient for this study is.87.

#### 2.2.4 The Depression Anxiety Stress Scale

The DASS assesses three negative emotion dimensions, namely depression (DASS-D), anxiety (DASS-A), and stress (DASS-S). Given that emotion dysregulation is implicated in the risk for depression, anxiety, and stress, this instrument was used as another important index of concurrent validity for DERS-SF. The Depression Anxiety Stress Scale (DASS) was developed by Lovibond in 1993 and consists of 42 items (33), all of which are negative emotional symptoms. Subsequently, in 1995 Lovibond updated DASS-21 into a shorter version (34) and Oei et al. further developed the instrument into DASS-18 in 2013 (35). Based on the study on Asian populations, including Malaysia, Indonesia, Singapore, Sri Lanka, Taiwan, and Thailand, it was discovered that after removing stress items from the stress scale, DASS-18 was found to have good internal validity in an Indonesian sample. With Cronbach’s alpha values of.87 for depression,.85 for anxiety, and.72 for stress, DASS-18 was considered to be more suitable for Asian populations (35). A Likert scale of 0 to 3 was used with choices ranging from “*Did not apply to me at all*” to “*Applied to me very much or most of the time*”. The higher the score, the greater the emotional anguish (34, 35). Furthermore, the Cronbach’s alpha coefficient for this study was.93.

### 2.3 Procedure

Initially, permission was requested from the author of DERS-SF, then two Indonesian bilinguals translated the original version into Bahasa Indonesia. Two more bilinguals further back-translated the Indonesian version into English and the back-translated version was compared to the original. Furthermore, experts were involved from the department of psychiatry, psychology, public health, linguistics, and clinicians. The comparison and consensus-seeking process adhered to the Guidelines for the Process of Cross-Cultural Adaptation Stage IV, known as the Expert Committee review based on the International Test Commission Guideline for Translating and Adapting Test (36). The team revised the questionnaire for any inaccuracies. A cognitive interview was also conducted with students (N = 48) before using the questionnaire as a tool. In addition, the questionnaire was modified when discrepancies were discovered.

Ethical approval was received from the Universitas Padjadjaran Research Ethics Committee (No.1135/UN6.KEP/EC/2020). At the beginning of the survey, participants were informed of voluntary withdrawal by simply closing the browser page. All of the information gathered was kept completely confidential.

Participants were recruited through announcements in schools, universities, and social networking services (SNS) during the specified period. All participants provided informed consent and completed the online survey through the SurveyMonkey app, which included the DERS-SF, the DASS, with depression inventory divided to adults participants (university students) filled the BDI-II, and adolescents participants (senior high school students) filled the CDI. This was supervised by the team using the Zoom video conference platform to detect the difficulties and side effects during the data collection. A retest was given to participants roughly two weeks after the initial data was obtained.

### 2.4 Statistical analysis

The mean and standard deviation were determined to describe the data. The groups of gender and education level were compared by T-test. The internal reliability of DERS-SF was assessed with Cronbach’s alpha. Cicchetti guidelines were used to interpret the Alpha score, i.e., a reliability coefficient below .40 indicates poor clinical significance, while a coefficient between .40 and .59 indicates fair clinical significance. A coefficient between .60 and .74 indicates good clinical significance, and a coefficient between .75 and 1.00 indicates excellent clinical significance (37). The test-retest reliability coefficient was calculated using Pearson product-moment correlation between the first- and second-time measures. Interpretation of the correlations coefficient are .10 to .30 = weak, .40 to .60 = moderate, .70 to .90 = strong, and 1.00 = excellent (38).

The content validity evidence of DERS-SF was measured using the content validity index (CVI) based on expert judgments. Concurrent validity with additional measurement methods such as the DASS, BDI-II for adults (for university students), and CDI for adolescent (for senior high school students) was assessed with Pearson product-moment correlation. Furthermore, confirmatory factor analysis (CFA) was used to evaluate the internal structure of DERS- SF and run by the LISREL 10.3 program. The results from Kaufman were used to create the four-factor models tested (4, 7, 20). CFA addresses some of the limitations in the exploratory factor model and is useful for evaluating the best fit of a model (39). The CFA model was evaluated using the adjusted goodness-of-fit index (AGFI), comparative fit index (CFI), non-normed fit index (NNFI), root mean squared error of approximation (RMSEA), and standardized root mean square residual (SRMR). Values for AGFI, CFI, and NNFI range from 0 to 1.0, with those greater than .90 implying a satisfactory fit to the data. Smaller values of RMSEA and SRMR also suggest a better fit, with values of .10 or less reflecting a good fit and.05 or less being considered a very good fit (40). Factor loadings of .32 or above were considered meaningful (41).

## 3 Results

### 3.1 Descriptive statistics

The means and standard deviations of DERS-SF subscales and other variables are summarized in **Table 1**.

**Table 1:** Mean and SD of the different subscales and total scores of DERS-SF, BDI-II, CDI, and DASS (N = 738).

	Mean	SD
DERS-SF Total Score	49.23	13.81
Awareness	7.47	2.69
Clarity	8.64	3.37
Nonacceptance	8.37	3.15
Goals	9.84	3.59
Impulse	7.42	3.67
Strategies	7.49	3.06
Beck Depression Inventory II Total Score	18.73	12.56
Cognitive	6.78	4.85
Affective	3.97	3.00
Somatic	7.98	5.59
Child Depression Inventory Total Score	14.51	7.36
Negative Mood	2.37	1.72
Interpersonal Difficulties	3.69	2.95
Ineffectiveness	2.90	1.79
Anhedonia	2.37	1.57
Negative Self-Esteem	3.20	1.34
Depression Anxiety Stress Scale		
Depression	4.98	4.56
Anxiety	7.03	4.73
Stress	4.08	2.70

SD, Standard Deviation.

### 3.2 Gender and education difference on DERS-SF subscales

The participants were divided into two education groups, senior high school (46.3%) and university students (53.7%). The gender and education level differences are summarized in **Tables 2** and **3**.

**Table 2:** Mean, standard deviation, and the t-test comparison among female (n = 567) and male (n = 170).

Scale	Male		Female		t_(df)_	P-value
	Mean	SD	Mean	SD		
DERS-SF total score	47.76	11.78	49.67	14.35	t_(333.28)_ = -1.76	.08
Awareness	7.54	2.59	7.44	2.72	t_(735)_ = .41	.68
Clarity	7.97	3.03	8.84	3.44	t_(311.56)_ = -3.17	.00
Nonacceptance	7.94	2.95	8.51	3.20	t_(298.47)_ = -2.17	.03
Goals	9.88	3.39	9.84	3.66	t_(296.47)_ = .13	.89
Impulse	7.45	3.52	7.41	3.73	t_(735)_ = .12	.90
Strategies	6.99	2.75	7.64	3.14	t_(312.81)_ = -2.59	.02

DERS-SF, Difficulties in Emotion Regulation Scale-Short Form; SD, Standard Deviation. From a total 738 participants, one participant did not provide gender data, resulting in a total of 737.

**Table 3:** Mean, standard deviation, and the t-test comparison among senior high school students (n = 342) and university students (n = 396).

Scale	Senior High School Students		University Students		t_(df)_	P-value
	Mean	SD	Mean	SD		
DERS-SF total score	49.47	13.82	49.03	13.81	t_(736)_ = .43	.67
Awareness	7.54	2.73	7.40	2.66	t_(736)_ = .70	.48
Clarity	8.76	3.48	8.53	3.26	t_(736)_ = .94	.35
Nonacceptance	8.38	3.12	8.37	3.19	t_(736)_ = .06	.95
Goals	9.56	3.71	10.09	3.48	t_(736)_ = -1.99	.05
Impulse	7.59	3.71	7.28	3.65	t_(736)_ =1.15	.25
Strategies	7.63	3.13	7.37	2.99	t_(736)_ = 1.18	.24

DERS-SF, Difficulties in Emotion Regulation Scale-Short Form; SD, Standard Deviation.

Statistically significant differences were identified between males and females for DERS-SF total score, clarity, non-acceptance, goals, and strategies subscales.

There were no statistically significant differences between senior high school and university students.

### 3.3 Reliability

Based on 738 participants, the result showed that DERS-SF had good internal consistency with Cronbach’s alpha coefficient of .87 for all 18 items. The internal consistency of the subscale clarity, goals, and impulse was considered good. While the non-acceptance and strategies subscale was fair. However, the internal consistency of awareness subscale was unacceptable. The corrected total item ranged from .26 to .82 as depicted in **Table 4**.

**Table 4:** Cronbach Alpha and corrected item total correlation of the DERS-SF subscales (n = 738).

DERS-SF Subscales	Item Number	Cronbach’s alpha	Corrected item total correlation
Awareness	1, 4, 6	.61	.26 –.52
Clarity	2, 3, 5	.87	.73 –.79
Non-acceptance	7, 12, 16	.70	.46 –.58
Goals	8, 11, 13	.89	.74 –.82
Impulse	9, 14, 17	.90	.80 –.82
Strategies	10, 15, 18	.72	.52 –.56

DERS-SF, Difficulties in Emotion Regulation Scale-Short Form; SD, Standard Deviation.

Based on the result that showed low reliability and low validity, we considered reanalyzing the result in 3 different ways. First, we reanalyzed all the original 18 items, resulting in Cronbach’s alpha coefficient of .89. Secondly, we deleted item number 6, which has low internal reliability and low corrected total item correlation. Cronbach’s alpha of 17 items is now .90. Lastly, we deleted the awareness subscale (items 1, 4, and 6). In this third alternative, the remaining 15 items resulted in Cronbach’s Alpha coefficient of .91.

The test-retest reliability was investigated using retest data acquired roughly two weeks following the initial measurement. The retest data were collected from 72.5% of the participants. The result of the test-retest reliability coefficient was .67, which is considered moderate.

### 3.4 Content validity

The content validity of DERS-SF was determined after evaluation by a panel of five experts from the departments of psychiatry, psychology, public health, and linguistics, showing that the instrument was relevant and representative. This was shown by a high item content validity index (I-CVI = .96) and scale content validity index (S-CVI = .83). In focus group discussions, participants found that DERS-SF was easy to understand and had satisfactory face validity. DERS-SF pilot tests have shown that the administrative time is acceptable and easy to understand.

### 3.5 Convergent validity

Evidence of convergent validity, were done by analyzing the correlation between DERS-SF and other criterion variables, such as DASS, BDI-II, and CDI. The BDI-II and CDI measure the symptoms of depression in adults and children, respectively. The DASS was created to measure negative emotions including anxiety, depression, and stress. This is consistent with the literature, stating that the scores for DERS are strongly correlated with psychopathology and inversely correlated with measures of psychological well-being (42).

**Table 5** shows that the DERS-SF total and subscales scores are significantly associated with the aforementioned questionnaires. All subscale scores had a positive correlation with DASS, BDI-II, and CDI except the awareness subscale.

**Table 5:** Correlation coefficient between DERS-SF and the criterion variables.

		DERS-SF						
		Total	Nonacceptance	Goals	Impulse	Awareness	Strategies	Clarity
DASS	Total	.67^**^	.47^**^	.52^**^	.57^**^	-.26^**^	.65^**^	.57^**^
	Depression	.55^**^	.40^**^	.43^**^	.44^**^	-.31^**^	.56^**^	.54^**^
	Anxiety	.61^**^	.45^**^	.47^**^	.51^**^	-.21^**^	.59^**^	.51^**^
	Stress	.66^**^	.42^**^	.53^**^	.62^**^	-.14^**^	.61^**^	.45^**^
BDI-II	Total	.62^**^	.42^**^	.49^**^	.49^**^	-.31^**^	.60^**^	.59^**^
	Cognitive	.64^**^	.47^**^	.50^**^	.50^**^	-.32^**^	.59^**^	.58^**^
	Affective	.55^**^	.35^**^	.43^**^	.42^**^	-.29^**^	.56^**^	.53^**^
	Somatic	.56^**^	.35^**^	.44^**^	.44^**^	-.27^**^	.54^**^	.55^**^
CDI	Total	.62^**^	.42^**^	.47^**^	.55^**^	-.32^**^	.67^**^	.59^**^
	Negative mood	.45^**^	.27^**^	.34^**^	.47^**^	-.19^**^	.45^**^	.40^**^
	Interpersonal difficulties	.57^**^	.38^**^	.43^**^	.45^**^	-.21^**^	.59^**^	.56^**^
	Ineffectiveness	.44^**^	.35^**^	.32^**^	.39^**^	-.39^**^	.53^**^	.46^**^
	Anhedonia	.50^**^	.35^**^	.38^**^	.47^**^	-.28^**^	.59^**^	.43^**^
	Negative self-esteem	.40^**^	.25^**^	.32^**^	.38^**^	-.20^**^	.42^**^	.36^**^

^**^ *p*< 0.01; DERS-SF, Difficulties in Emotion Regulation Scale-Short Form; DASS, Depression Anxiety Stress Scale; BDI-II, Beck Depression Inventory-II; CDI, Children Depression Inventory.

### 3.6 Factorial validity

**Figure 1:**
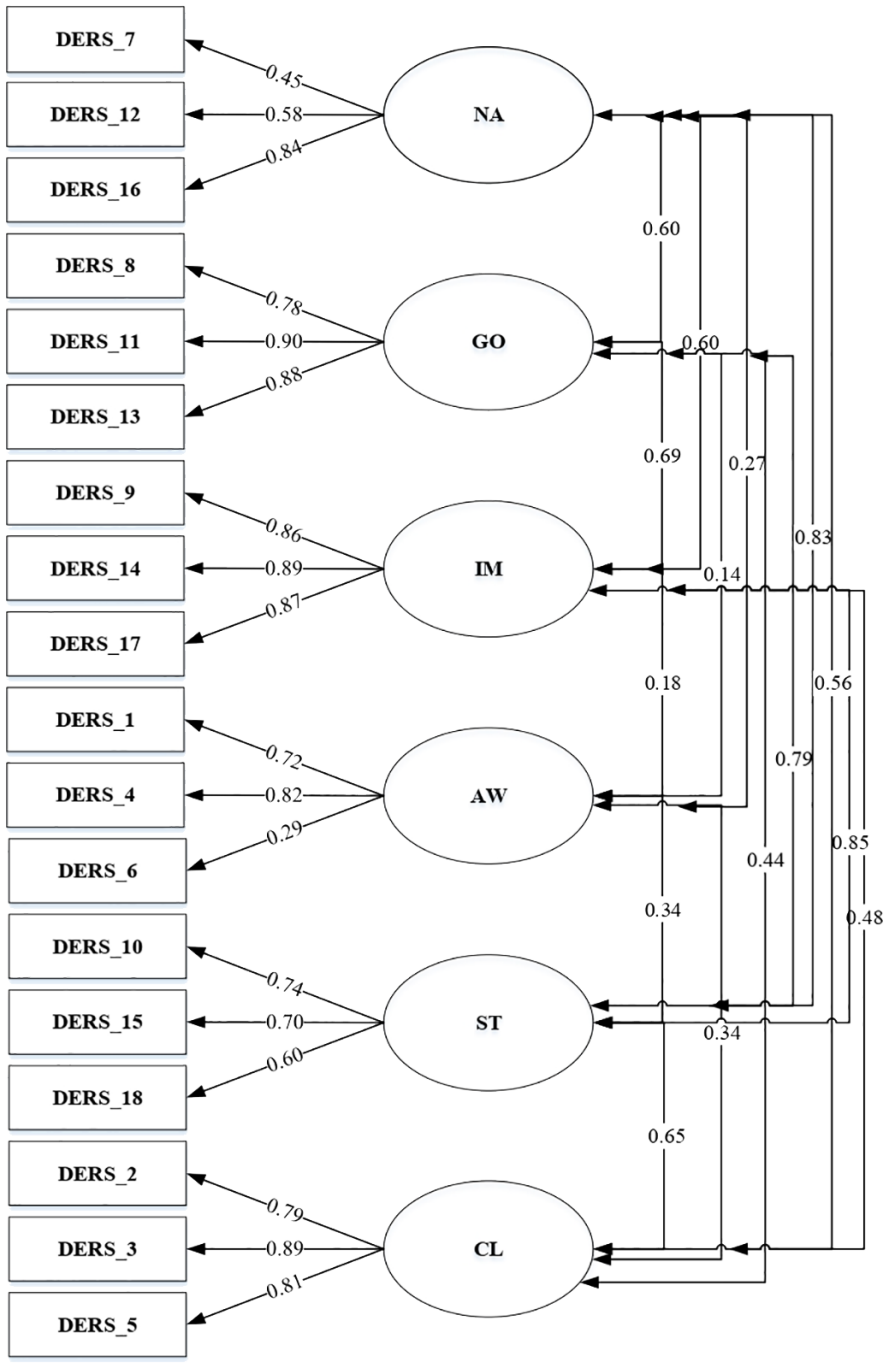
DERS-SF Correlated 6-factor model (Model 1).

**Figure 2:**
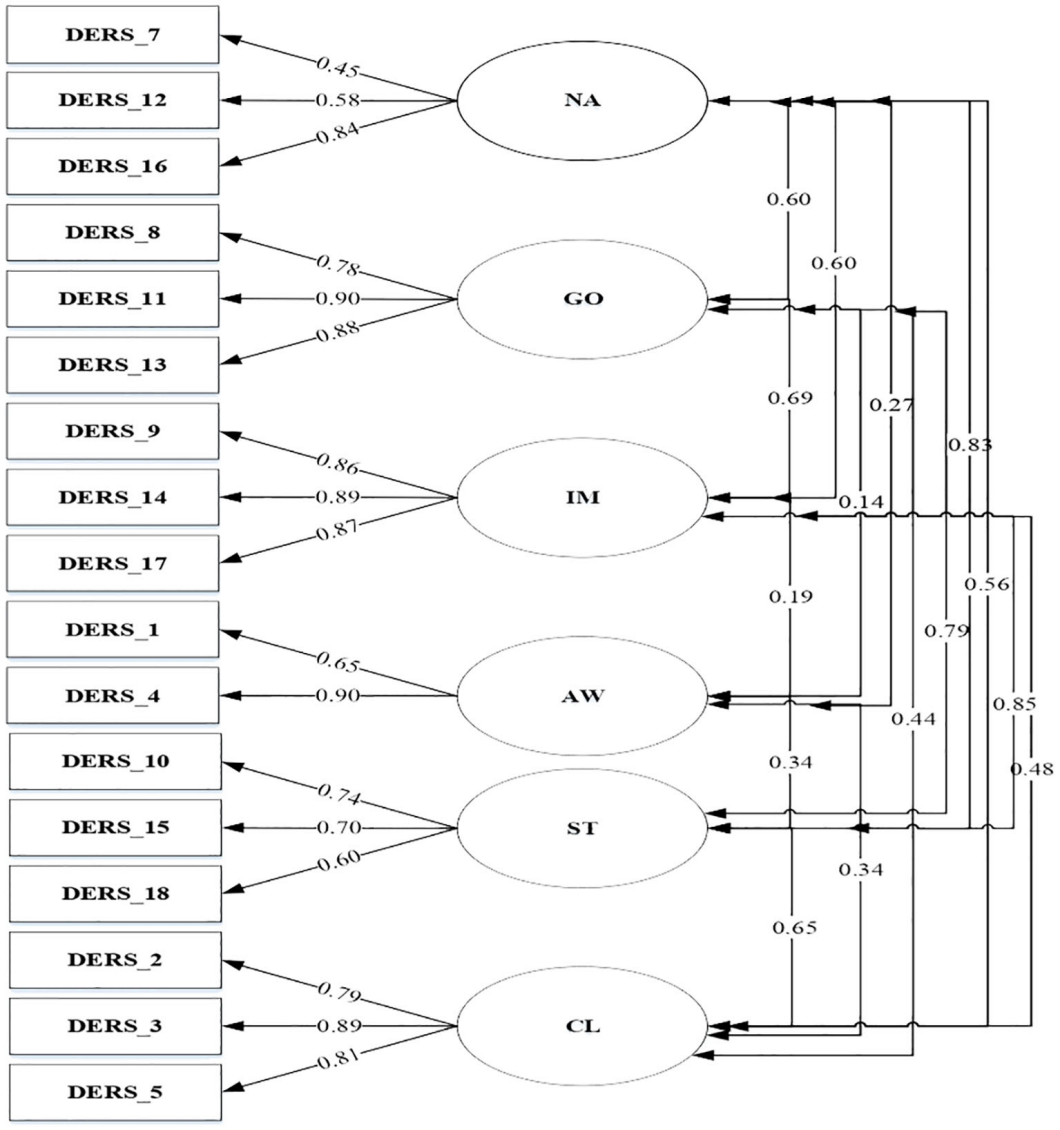
DERS-SF Correlated 6-factor model excluding item number 6 (Model 2).

**Figure 3:**
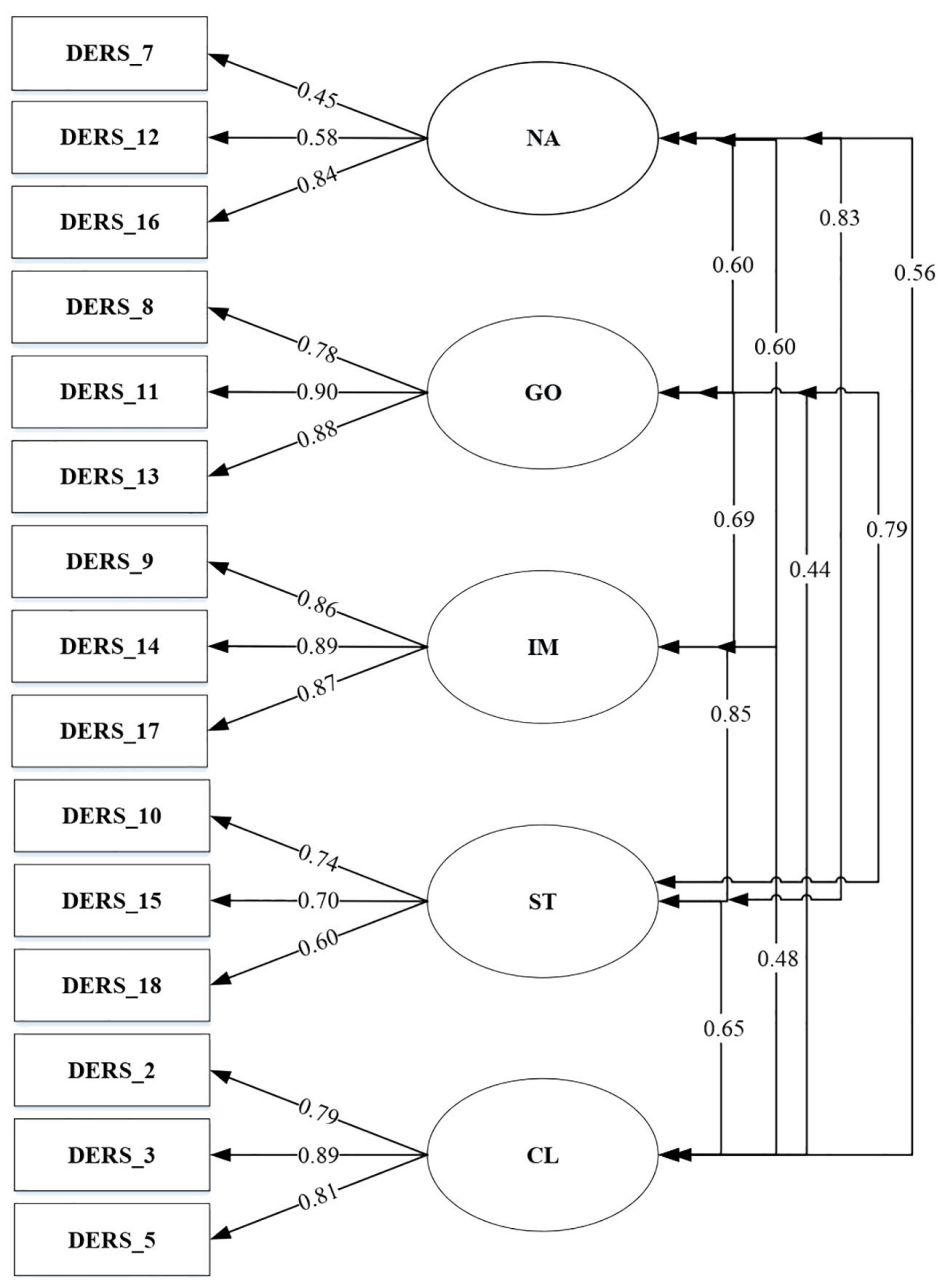
DERS-SF Correlated 5-factor model excluding awareness (Model 3).

Kaufman’s six-factor model, which permitted correlation between all DERS-SF aspects was tested (Model 1), presenting a good fit to the data. As presented in **Figure 1**, all items in model 1 loaded significantly on the general factor, except item number 6 (factor loading .29). Based on the results, **Figure 2** depicted a factor model that was constructed without item 6 (Model 2). Moreover, since several studies failed to confirm the stability and reliability of the awareness subscale, this study also developed a factor model without the awareness subscale (Model 3) as seen in **Figure 3** (17, 20, 43).

A total of three models were tested through CFA. The goodness of fit indices of the factor model is shown in **Table 6**.

**Table 6:** The goodness of fit indices of the factor model.

Model factors	χ^2^	df	χ^2^/df	AGFI	NNFI	CFI	RMSEA	SRMR
Model 1 (Correlated 6-factor model)	418.85	120	3.49	.91	.95	.96	.06	.05
Model 2 (Correlated 6-factor model excluding item number 6)	360.23	104	3.46	.92	.95	.96	.06	.04
Model 3 (Correlated 5-factor model excluding awareness)	331.10	80	4.14	.91	.95	.96	.07	.04

AGFI, adjusted goodness-of-fit index; NNFI, the non-normed fit index; CFI, comparative fit index; RMSEA, root mean squared error of approximation; SRMR, standardized root mean square residual.

## 4 Discussion

This study aimed to translate and adapt DERS-SF into Bahasa Indonesia, evaluate the factor structure, and investigate the psychometric properties among high school and university students. The results showed that the mean average total score of DERS-SF among the participants was 49.23, surpassing the value obtained in previous studies. Gouveia et al. found that the mean total score of DERS-SF among adults was 38.59 (44), while the values obtained by Kaufman et al. were 40.32 and 36 for adolescents and university students, respectively. Additionally, Moreira et al. found that the mean scores among adolescents and adults were 43.2 and 37.26. Eloranta et al. also reported a mean score of 37.62 among adolescents (7, 20, 43).

The higher mean score of the DERS-SF total could be attributed to data collection during the COVID-19 pandemic. This period is characterized by unprecedented national and global health, social, and economic emergencies, which may influence emotion. Besides, higher levels of emotion dysregulation were associated with greater boredom proneness during the COVID-19 pandemic, which restricted social interaction among students and caused significant lifestyle changes (45).

There were significant differences in non-acceptance, strategies, clarity, and goal scores between males and females. The female scores were higher than the male for clarity, non-acceptance, and strategies, while males had greater scores for goals. This result was in line with Veloso and Shahabi, stating the clarity subscale was significantly different among the Portuguese (46), Persian (47), and Finnish population (43). However, there were inconsistencies. According to Veloso, men showed more difficulties recognizing emotion, while Shahabi and Eloranta found more difficulties in women. Studies by Gouveia et al. (2022) (44) carried out on the Portuguese population, and Eloranta et al. (2020) (18) on the Finnish population found differences in the strategies subscale with females scores higher than males. The non-acceptance subscale significantly differed among both genders in the Finnish population (43), with higher female scores than males. Females find it more difficult to regulate emotion due to gender disparities in self and environmental perceptions (48). Research from Alhadi et al. (2019) in Indonesia show that females manage and regulate their emotions better in certain situations (49). Females are more likely to control their anger and emotions in order to avoid maladaptive behavior and aggression, also that counseling services can help improve these skills (50).

The results showed that DERS-SF has good internal consistency, except for awareness subscale, with Cronbach’s alpha coefficient a bit lower than.70. The corrected item-total correlation was considered moderate to strong, except for awareness item number 6. Furthermore, the convergent validity of the questionnaire was observed by elaborating on the associations between DERS-SF with DASS, BDI-II, and CDI. All the subscale scores are strongly correlated with DASS, BDI-II, and CDI except the awareness subscale, which ranged between -.39 and -.19. Based on the results, there was a weak negative correlation between the awareness subscale and symptoms of stress, anxiety, and depression. These outcomes were consistent with Salters-Pedneault et al. stating that only awareness in the DERS subscales failed to significantly predict the diagnostic status of GAD. Neumann et al. also reported that awareness did not correlate with anxiety and depression (51, 52). An awareness of emotional experiences does not always imply a healthy response to or regulation of such states (53). Although some forms of emotional awareness may be adaptive, such as non-judgemental acceptance, Tull et al. reported that other forms are probably maladaptive, including rumination on negative emotion (54).

Study from Bhatnagar et al. consistent with Bardeen et al. suggested that one possible issue with the Awareness domain is the way the construct is operationalized. in these studies the Awareness items in the DERS-SF are reverse-keyed, which led to negative correlations not observed in other subscales of the DERS-SF and measures such as BDI-II, CDI, and DASS that shown in this study result (53, 55).

In general, all items had significant factor loadings with moderate to high coefficients, except item number 6 (with factor loading .29). Similar results were reported by Gouveia among Portuguese with a factor loading of.39 and Asgarizadeh among the Iran population, which obtained values of.33 and.42 in community and students, respectively (44). However, item number 6 in the United States presented strong factor loadings for adolescents (.73 and .71) as well as college students (.63 and .69) (7, 56). In this study, item number 6 was removed, but the factor loadings in models 2 and 3 showed significant results with coefficients ranging from .45 to .90. The statement in item number 6, “*When I’m upset, I acknowledge my emotion*” shows awareness and understanding of emotion. The ability to recognize emotion can be influenced by culture. Culture plays a significant role in determining whether individuals are motivated to regulate their emotions and whether such regulation is adaptive or maladaptive. Nuanced cultural analysis can enhance our comprehension of emotion regulation. For example, the concept of understanding emotion in Indonesia could be different from American or other Western cultures (57). Cultural backgrounds and situational demands interact to shape how people regulate positive emotions, with European Americans tending to savor positive emotions more in high cognitive effort contexts than Asians (58). Interpersonal emotion regulation strategies, such as social modeling and perspective taking, may be more beneficial for East Asian groups in reducing negative affect during stressful situations (59). Emotion regulation strategies improve mental health across cultures, but masking negative emotions out of concern for others is associated with better mental health in Japanese (60).

A total of three models were tested through CFA. The correlated 6-factor model (model 1), 6-factor model excluding item number 6 (model 2), and 5-factor model excluding awareness (model 3) presented good fit data. This result was in line with another study that tested CFA among the Spanish, Portuguese, United States, and Iranian populations (17, 20, 44, 56, 61). The reliability result showed that when item number 6 was removed from the analysis, it has better internal reliability and CFA validity. This is in line with removing the three items in the awareness subscale, which leads to better Chronbach’s Alpha coefficient and validity. However, removing the awareness subscale from DERS-SF might needed some consideration from the theoretical aspect of emotion regulation.

The awareness subscale might assess a different aspect of emotion regulation from the other subscales. Awareness acts in the ability to control and understand emotion, whereas this process happens at an earlier stage of emotion regulation. Meanwhile, the other subscale occurs in the later stage, where an individual focuses on the strategy to control their response to the emotion, as mentioned in the “modal model” of emotion (62). This theory explains why the awareness subscale is considered to measure a different construct (20).

The main limitation of this study was that the data were administered to students during the COVID-19 detention, a situation of unprecedented national and global health, social, and economic emergency. Another limitation was that the results could not be extrapolated across age groups or to clinical populations in Indonesia because the study solely focused on young adults and adolescents.

## 5 Conclusion

In conclusion, this is the first study to adapt DERS-SF to the Indonesian-speaking population. The instrument showed good internal consistency and stability over time (test-retest). In addition, the content and convergent validity were proven acceptable in non-clinical settings. Models 1, 2, and 3 were proposed to be good tools for assessing the difficulties of emotional regulation among adolescents and adults. However, our findings suggest that model 3, where the awareness subscale is removed, has better psychometric properties. Therefore, removing the awareness subscale might be a better fit after adapting the Indonesian version of the DERS-SF, mainly for the non-clinical population. Future studies could focus on additional investigations among the Indonesian clinical population.

## Data availability statement

The raw data supporting the conclusions of this article will be made available by the authors, without undue reservation.

## Ethics statement

The studies involving humans were approved by Universitas Padjadjaran Research Ethics Committee. The studies were conducted in accordance with the local legislation and institutional requirements. Written informed consent for participation in this study was provided by the participants’ legal guardians/next of kin.

## Author contributions

FD: Methodology, Writing – review & editing, Writing – original draft, Formal Analysis, Conceptualization. VP: Writing – review & editing, Methodology, Funding acquisition, Formal Analysis, Conceptualization. EF: Writing – review & editing, Validation, Software, Methodology, Formal Analysis. IA: Writing – review & editing, Formal Analysis, Conceptualization. FP: Writing – review & editing, Methodology, Conceptualization. AI: Writing – original draft, Project administration, Investigation, Data curation. KP: Project administration, Writing – original draft, Investigation, Data curation. AS: Writing – original draft, Resources, Project administration, Data curation. FM: Investigation, Writing – review & editing, Writing – original draft, Project administration, Data curation. RE: Investigation, Writing – original draft, Project administration, Data curation.

## Supplementary material

Data Sheet 1 Indonesian version of DERS-SF.

Data Sheet 2 English version of DERS-SF.
