# Supplementary material for: Validity and reliability of the Difficulties in Emotion Regulation Scale Short Form in Indonesian non-clinical population
Source: Front Psychiatry. 2024 Mar 25;15:1380354. doi: 10.3389/fpsyt.2024.1380354 (PMC11000630; doi:10.3389/fpsyt.2024.1380354)
Supplement: Data Sheet 1 — Indonesian version of DERS-SF. [file DataSheet_1.pdf]

Difficulties In Emotion Regulation Scale -Short Form (DERS-SF)

Tolong tunjukkan seberapa sering pernyataan berikut ini berlaku untuk Anda dengan menuliskan angka yang sesuai dari skala di bawah pada baris di samping setiap item.

No	Indonesia	(1) Hampir tidak pernah 0-10 %	(2) Terkadang 11 – 35 %	(3) Sekitar separuh waktu 36-65 %	(4) Sebagian besar waktu 66 – 90 %	(5) Hampir selalu 91 – 100 %
1	Saya memberikan perhatian terhadap perasaan saya					
2	Saya tidak tahu yang sedang saya rasakan saat ini					
3	Saya sulit memahami perasaan saya					
4	Saya peduli akan perasaan saya					
5	Saya bingung dengan yang sedang saya rasakan					
6	Ketika kesal, saya mengakui perasaan kesal tersebut					
7	Ketika kesal, saya menjadi malu karena perasaan kesal tersebut					
8	Ketika kesal, saya sulit menyelesaikan pekerjaan					
9	Ketika kesal, saya menjadi tidak terkendali					
10	Ketika kesal, saya yakin bahwa pada akhirnya saya akan merasa sangat tertekan					
11	Ketika kesal, saya sulit fokus pada hal lain					
12	Ketika kesal, saya menjadi merasa bersalah karena perasaan kesal tersebut					
13	Ketika kesal, saya sulit berkonsentrasi					
14	Ketika kesal, saya kesulitan mengendalikan perilaku saya					
15	Ketika kesal, saya yakin tidak ada yang bisa saya lakukan untuk membuat diri saya merasa lebih baik					
16	Ketika kesal, saya menjadi jengkel pada diri sendiri karena perasaan kesal tersebut					
17	Ketika kesal, saya kehilangan kendali terhadap perilaku saya					
18	Ketika kesal, saya butuh waktu lama untuk merasa lebih baik					
