# Supplementary material for: Validity and reliability of the Difficulties in Emotion Regulation Scale Short Form in Indonesian non-clinical population
Source: Front Psychiatry. 2024 Mar 25;15:1380354. doi: 10.3389/fpsyt.2024.1380354 (PMC11000630; doi:10.3389/fpsyt.2024.1380354)
Supplement: Data Sheet 2 — English version of DERS-SF. [file DataSheet_2.pdf]

Difficulties in Emotion Regulation Scale – Short Form (DERS-SF)

<u>Please indicate how often the following apply to you.</u>

No.		Almost Never (0–10%)	Some-Times (11–35%)	About Half Of the Time (36–65%)	Most of the Time (66–90%)	Almost Always (91–100%)
1	I pay attention to how I feel	1	2	3	4	5
2	I have no idea how I am feeling	1	2	3	4	5
3	I have difficulty making sense out of my feelings	1	2	3	4	5
4	I care about what I am feeling	1	2	3	4	5
5	I am confused about how I feel	1	2	3	4	5
6	When I'm upset, I acknowledge my emotions	1	2	3	4	5
7	When I'm upset, I become embarrassed for feeling that way	1	2	3	4	5
8	When I'm upset, I have difficulty getting work done	1	2	3	4	5
9	When I'm upset, I become out of control	1	2	3	4	5
10	When I'm upset, I believe that I will end up feeling very depressed	1	2	3	4	5
11	When I'm upset, I have difficulty focusing on other things	1	2	3	4	5
12	When I'm upset, I feel guilty for feeling that way	1	2	3	4	5
13	When I'm upset, I have difficulty concentrating	1	2	3	4	5
14	When I'm upset, I have difficulty controlling my behaviors	1	2	3	4	5
15	When I'm upset, I believe there is nothing I can do to make myself feel better	1	2	3	4	5
16	When I'm upset, I become irritated with myself for feeling that way	1	2	3	4	5
17	When I'm upset, I lose control over my behavior	1	2	3	4	5
18	When I'm upset, it takes me a long time to feel better	1	2	3	4	5
